# Calibration of an Autonomous Instrument for Monitoring Light Pollution from Drones

**DOI:** 10.3390/s19235091

**Published:** 2019-11-21

**Authors:** Pietro Fiorentin, Carlo Bettanini, Damiano Bogoni

**Affiliations:** Department of Industrial Engineering, University of Padova, 35100 Padova, Italy; carlo.bettanini@unipd.it (C.B.); dambog@hotmail.it (D.B.)

**Keywords:** imaging system, image luminance measuring device, light pollution, artificial lighting, urban analysis, remote sensing, photometry, spectral analysis, UAV

## Abstract

The paper presents the calibration activity on the imaging system of the MINLU instrument, an autonomous sensor suite designed for monitoring light pollution using commercial off-the-shelf components. The system is extremely compact and with an overall mass below 3 kg can be easily installed as a payload for drones or sounding balloons. Drones and air balloons can in fact play an important role in completing upward light emission measurement from satellites allowing an increased spatial and time resolution from convenient altitudes and positions. The proposed system can efficiently measure the luminous intensity and the spectral power density of on-ground emissions providing a useful tool to identify polluting sources and to quantify upward light flux. The metrological performance of the imaging system has been verified through an extensive laboratory test activity using referenced light sources: the overall uncertainty of the multi-luminance meter has been calculated to be 7% of the reading, while the multi-spectrometer has shown a full width at half maximum (FWHM) equal to 10 nm within the measuring range between 400 nm and 700 nm. When operating at an altitude of 200 m, the system can achieve a horizontal resolution at a ground level of 0.12 m with a wavelength resolution able to identify the different lamp technology of outdoor light sources, including light-emitting diode (LED) lights that are undetected by satellites.

## 1. Introduction

In recent decades, both the growth of wellbeing and the increasing request of safety, along with technological improvements in light sources, in particular in the luminous efficiency of LEDs [1], has driven the request of new lighting systems along motorized roads, cycle tracks, pedestrian zones, roads parking lots, and working places [2]. Unfortunately, part of the light directly emitted by outdoor sources and part of the light reflected by lit objects go towards the sky. A portion of that is redirected downwards and a diffuse artificial sky brightness is seen; it is called skyglow [3,4,5]. The artificially lit night has several negative effects on the environment, on plants, on animals and on people [6], with the most evident consequence in the loss of visible stars due to contrast reduction [7]. Light polluted skies are seen by more than 80% of people in the word, and they prevent more than one third of the world population from admiring the Milky Way, our Galaxy [8]. It was proven that being exposed for a long time at night to artificial light has a significant effect on the environment, wildlife, and society; furthermore, it also disturbs human health and sleeping rhythms [9,10].

The request of decreasing energy consumption to preserve the environment is driving to a wide-scale change in the used lighting technology, switching to LEDs [11] as a source for street lighting [12,13]. Unfortunately, LEDs are characterized by emission at the shortest wavelengths in the visible range, which are particularly unhealthy for humans [9] and the environment [14,15] and can alter stars observation [16]. The awareness of such problems is leading the design of eco-friendly spectral composition of lamps, but, in the meantime, effective approaches to mitigate the artificial light at night can be decreasing the intensity and the duration of outdoor lighting, reducing the trespass of lighting [17] and the creation of protected areas like national and international dark sky nature reserves [18].

Measurements of sky brightness were performed to evaluate the entity of light pollution [3]. The activity was organized by professional astronomers, amateur associations, and private citizens. Ground based inspections were also carried out, but many efforts are required to get a big sample of data [19]. This way is heavy for creating maps covering wide areas and tracking variations of the light emission.

Images from Earth-observing satellites have also been used to evaluate upward light emission from extended areas. Most of the used satellites are part of the Defense Meteorological Satellite Program, and their sensors did not perform a radiometric calibration before launch [20]. Sequent in-flight calibration based on statistical data and inter-calibration methods had anyway allowed for highlighting an increase in global lighting [21,22,23]. Some common issues may be underlined: images are affected often by saturation when observing cities due to their high upward luminous flux, low resolution in the signal acquisition (8 bits), and low sampling rate cannot track the dynamics behavior of different observed regions of Earth. Furthermore, observation from satellites has a horizontal spatial resolution of 5 km [24]. However, a significant correlation was highlighted by the analysis of satellite observations between human pressure on the environment and artificial light at night [25,26].

The Visible Infrared Imaging Radiometer Suite Day-Night Band (VIIRS DNB) [24] brought a new opportunity in the analysis of upward emitted light; in fact, it improves the horizontal resolution to 0.6 m and its wavelength responsivity is between 500 nm to 900 nm, achieving enough sensitivity to detect the light emitted from a single luminaire. The dynamic response is improved, and the data are digitized at 14 bits. It is a useful tool to identify major sources of upward light, some of them are airports and road-rail distribution hubs. Data from that satellite allow for finding different light footprints upon nighttime of cities and nations in terms of light management [27,28].

In any case, data from satellites present limitations. The spectral response of the radiometric sensor does not allow for discriminating “white” light sources. The nowadays transition from low pressure sodium to LED lighting makes this ability particularly useful. The limit at the lower wavelengths of their sensor responsivity causes difficulties in managing new light sources, which presents a rise of the light components at the shortest wavelengths. Satellite observations are therefore not able to describe the dynamic change of outdoor lighting. Furthermore, the satellite overpass is variable and asynchronous with variations of outdoor lighting. Another problem is due to the fact that photos collected from satellites are taken at nadir, while a small fraction of light is directly emitted upwards, in particular from newer light sources. Conversely, it is of interest to know how the emission varies with the observing angle.

Aerial observations can provide data from closer view than satellites [29,30,31]. The spatial resolution can be about 10 m, which can be enough for light pollution analysis. The images are acquired by color, red, green and blue (RGB), or monochromatic cameras; in the last case, wide pass-band filters are used to divide the visible spectrum in three wide bands, which are considered enough to identify most types of street lamps. Aerial surveys also perform measurements of light emission at the zenith of the sources, limiting the possibility of quantifying light emitted in any direction and at any wavelength [27,28].

Acquisitions from drones and air balloons allow for improving terrestrial surveys overcoming limits of data from satellites and aerial observations. The site is usually observed from a reduced height, achieving good spatial resolution and allowing at the same time use of lens with wide angle of view, which can collect light in directions far from the optical axis. The same site can be observed for an extended period monitoring variation of the luminous flux or instrumentation may be moved for easy repositioning of the flying system and spanning a quite wide area. In particular, unmanned aerial vehicles were already proposed to assess light pollution by measuring the distribution of luminous flux emitted by sports facilities [32]. The suggested method of light measurement is based on a goniometric system, the unmanned aerial vehicle moves in the three-dimensional space around the analyzed source bringing a photometer mounted on a gimbal. 

The measuring system (MINLU) presented in this work was designed as an autonomous sensor suite able to analyze the global upward luminous emission of outdoor lighting [33,34], identifying light polluting sources and characterizing them by a direct measurement of their spectral power density, i.e., source technology. MINLU was made by using low cost, easy to buy, commercial components; therefore, it can be well reproduced by any laboratory. The counterpart is the need of a devoted calibration, which is presented here. It presents a simple structure and a simple realization of the main instruments: the image luminance meter and the multi-spectrometer. The system is compact, its occupied space is a few cubic decimeters, and is light weight, less than 3 kg. Therefore, it does not present problems related to carrying it. The limits on the area to be analyzed are only due to flight constraints and safety authorizations. Apart from those limits, the system can fly over any site, in particular artificially lit sites where outdoor lighting systems can be easily identified from the collected images. The 4K resolution of the images of the studied areas, collected from hundreds of meters, allows a clear identification of framed objects, in particular polluting light sources. The wide angle of view of the used cameras also gathers light in directions far from the optical axis (nadir of the MINLU system), the more harmful ones. MINLU presents the ability of staying operating for hours, which is a quite long time in light pollution analysis. Staying over the site, the system held by a tethered balloon can record the dimming of outdoor lights during the night analyzing the effect of its evolution. That result can not be obtained from satellite observations and aerial overflights, which can only pass through and collect few images corresponding to a very short time interval during the night. Furthermore, the use of a drone allows for recording detailed images even of areas not well covered by images from satellites. All these results and actions provided by MINLU will complete the measurements of upward light emission obtained from satellites.

## 2. Materials and Methods

**Figure 1:**
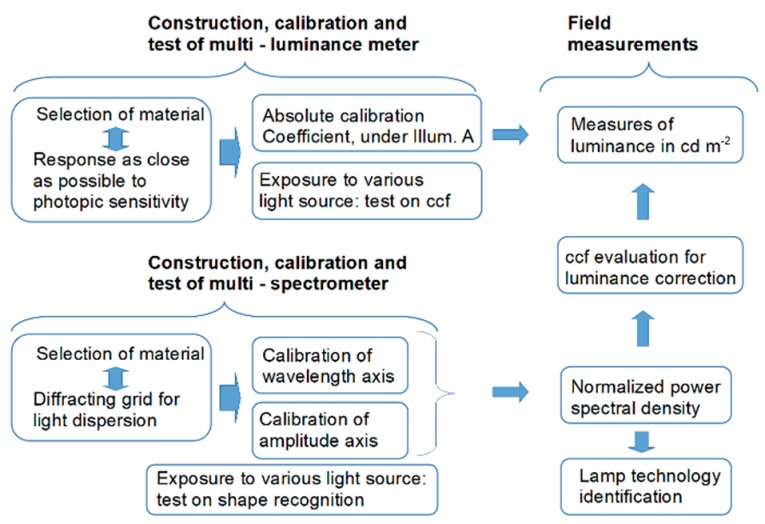
Diagram of the methodology steps.

The polluting sources framed by the proposed sensor suite are analyzed by measuring their luminous intensity of and their spectrum. Luminous intensity values are derived by measuring the luminance of light sources and their apparent emitting area. Those quantities are measured by an image luminance measuring device. For a better identification, the spectra of the different sources are also measured by an image device. In the construction of those instruments, commercial off-the-shelf products were used. Therefore, the system requires a calibration, and the methodology is briefly sketched in Figure 1. For the luminance meter, a response as close as possible to the photopic sensitivity and an absolute calibration are desired. The wavelength resolution of the spectral measures shall allow for distinguishing among the different lighting technologies; this requirement brings the choice of the dispersive element. Consequently, a calibration of the wavelength and amplitude axes is required. The measures from the multi-spectrometer will be used to correct the measured luminance values.

### 2.1. The Instrument Architecture

A Central Data Management Unit (CDMU) completely handles the instrument. This unit includes all electronics required for sensor conditioning, acquisition, compression, and storage of data. CDMU manages the imaging subsystem, which in the baseline flight configuration includes three digital cameras. A Stellarnet Black Comet spectrometer (Tampa, FL, USA) may optionally be added to measure the total upward spectral irradiance using a dedicated USB interface.

A Raspberry PI3 supports the CDMU, which is realized by a custom software based on Linux OS running on that board.

A high capacity 5S Lithium Polymer (Li-Po) battery provides the power to the system. The stored energy is enough for 4 h of continuous operation, it is delivered by a Power Distribution Unit with stable voltages to all subsystems. The payload composed by the instruments and their power supply weighs about 2.5 kg, and the contribution of the battery is about 0.2 kg. The drone weighs about 8 kg, and the power required to fly is provided by an extra battery which assures a flight duration of about 25 min. That time is considered enough for the inspection of an area. The 4 h of continuous acquisition of the three cameras, the position, pointing, and telemetry devices are useful when MINLU is held by a tethered balloon. This long time allows the system to analyze a significant part of the night, if required by segmenting the acquisition, and to record the dimming of outdoor lights.

**Figure 2:**
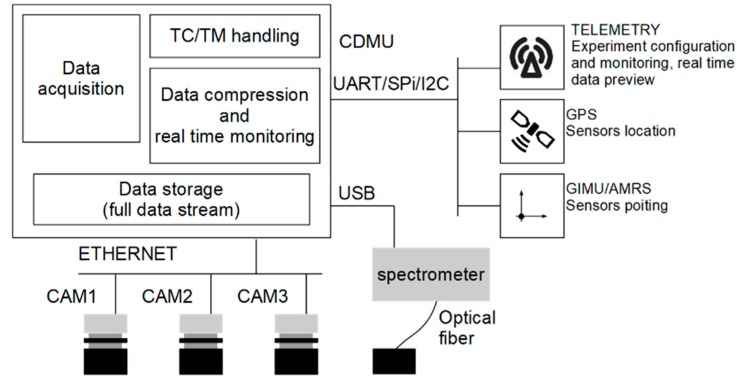
Sketch of MINLU architecture.

Housekeeping sensors, environmental sensors, inertial measurement unit (IMU), and global positioning system (GPS) are also present to monitor system operation and allow image georeferentiation. A sketch of the realized architecture is presented in Figure 2.

An on-board timer synchronizes and triggers the data-acquisition. The position of the system and the pointing retrieved by the GPS and Attitude and Heading Reference System (AHRS) are correlated with the acquired data. An on-board non-volatile memory stores the acquired data, which will be processed later on ground. A telemetry link allows communication between the CDMU and ground. Telecomands (TC) sent from ground can be used to configure the acquisition parameters. During the experiment, the CDMU generates in real-time quick-look data, and they are sent to the ground by Telemetry (TM). Those data include properly decimated and compressed images. On the basis of this information, the user can check acquired data and consequently perform fine-tuning of the experiment.

### 2.2. The Imaging Subsystem

The imaging subsystem relays on three digital cameras from Basler (Ahrensburg, Germany): two are of type acA3088-16gm, with a monochromatic complementary metal–oxide–semiconductor (CMOS) sensor (Sony STARVIS CMOS IMX178LLJ-C) (Tokyo; Japan) and one is a model acA3088-16gc, with a color CMOS sensor (Sony STARVIS CMOS IMX178LQJ-C) (Tokyo; Japan). Sensor size is 7.4 mm × 5 mm with a resolution of 3088 horizontal pixels × 2064 vertical pixels and uses a back-illuminated technology improving low-light performance and overall sensitivity. The detailed camera’s specifications are presented in Table 1. 

A Theia Technologies ML410M manual varifocal lens (Wilsonville, OR. USA) is used in front of each of the three cameras: the settable focal length from 4 mm to 10 mm allows for varying the angle of view of the system up to 90°; every camera system can therefore analyze upward emitted rays that are away from the nadir direction up to 45°, greatly enhancing opportunities in the study of upward-emitting light sources, particularly with respect to satellite measurements. Furthermore, by moving the acquisition system horizontally, the same light source can be observed from several directions away from its zenith, measuring the luminous intensity of polluting sources in the most harmful directions. A ground resolution better than 1 m can be easily obtained flying at low altitude as payload of drones or balloons: at a flying altitude of 200 m, the framed surface is 360 m × 240 m achieving a horizontal resolution at ground level around 0.12 m. The measurements of luminance, luminous intensity and spectrum of street luminaires and other polluting light sources could be affected by humidity in particular at angles far from nadir of the measuring system. Up to now, outdoor tests were done only in dry conditions, and no correction factor was required.

**Figure 3:**
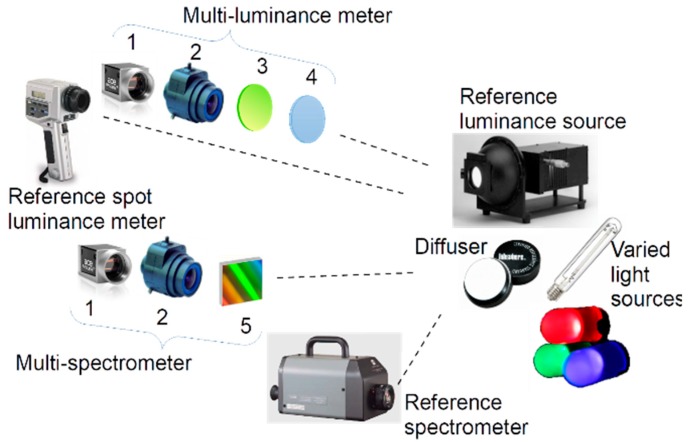
Sketch of the materials and of the test rig used for the calibration of the multi-luminance meter and the multi-spectrometer: (**1**) is the commercial camera, (**2**) is the lens, (**3**) is the photopic filter, (**4**) is the NIR low pass filter, (**5**) is the transmission diffracting grid.

The two monochromatic cameras use a dedicated optical filter and a visible transmission diffracting grid in front of the lens to work respectively as a multi-luminance meter and, as a raw multi-spectrometer, while the RGB camera is used to document the framed scene. It has to be noted that, during operation, the system is designed to analyze emissions from sources far away from the optics; therefore, all rays collected by the frontal lens and coming from a point in the framed scene can be supposed having the same direction. The deviation of the rays caused by a frontal filter has therefore a negligible effect. On the contrary, the rays converging on a pixel of the sensor after they have passed through the lens have different directions and, as a result, their refraction caused by a filter placed near the sensor would deteriorate the focus of the lens. This was the main driver in the design of the optical system with the filters placed in front of the lens. The components are sketched in Figure 3.

**Figure 4:**
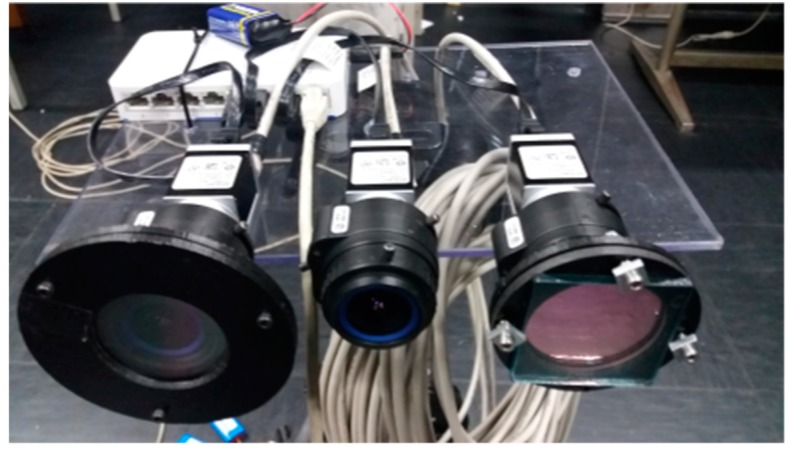
The multi luminance meter (on the right) and the multi-spectrometer (on the left); the color RGB camera is in the center.

A picture of imaging subsystem comprising the RGB camera, the multi-luminance meter, and the multi-spectrometer is presented in Figure 4 showing the CMOS cameras, the lenses, the photopic filter of the multi-luminance meter, and the diffracting grid of the multi-spectrometer.

**Figure 5:**
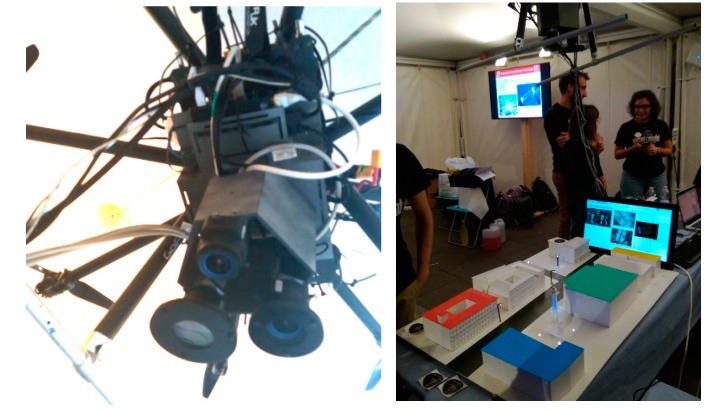
MINLU system on octocopter drone and Autonomous system for control operation with mockup of street illumination.

The whole MINLU instrument has been installed as a payload on an octocopter drone and the autonomous operation of the whole flying assembly has been verified in laboratory with a mock-up of street illumination and with drone test flights (Figure 5).

### 2.3. The References for the Calibrations

The calibrations of the multi-luminance meter and the multi-spctrometer are obtained by comparison of the MINLU components with a conventional spot luminance meter, Konica Minolta LS-100 (Tokyo, Japan), and a commercial spot spctrometer, Konica Minolta CS-1000. They are sketched in Figure 3 observing a reference luminance source and a diffuser lit by varied light sources used during the calibrations. The characteristics of the reference instruments are described in detail in the section devoted to the calibration.

## 3. Calibration of the Imaging Subsystem of the MINLU Instrument

The camera assemblies (camera, lens, and filter) operating as a multi luminance meter and multi-spectrometer have been calibrated in the Photometry and Light Engineering laboratory at University of Padova comparing the response to different inputs with reference commercial instruments. 

**Figure 6:**
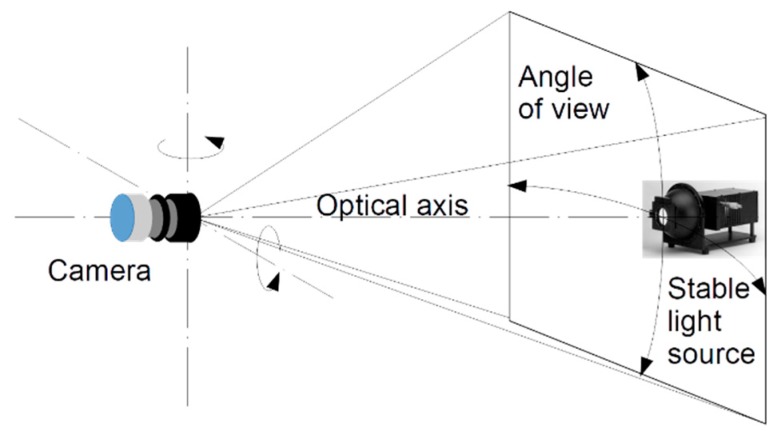
Geometry used to compensate the vignetting: the camera revolves of around both axes orthogonal to its optical axis observing a stable light source.

Due to the wide view angle of the lens system, a vignetting effect [35] was expected for all cameras, i.e., a reduction of the image’s luminance, with respect to luminance of the framed surfaces, going towards the periphery of the sensor from the image center. The effect is usually corrected by using a dedicated calibration procedure, which requires placing each camera with its optics on a goniometer [36], to expose the system to a reference source with a known stable luminance and to revolve the camera around both axes orthogonal to its optical axis, scanning any lighting direction, according to the procedure described in [37,38]. A sketch illustrating the revolving of the camera is presented in Figure 6. The illuminance reduction in the directions far from the optical axis can be elaborated to build up a correction matrix, which describes the vignetting effect. In this work, a simpler approach based on the experience gained on similar optical systems was implemented: an analytical function was obtained by fitting experimental data on few illuminating directions and used to calculate the correction matrix. This approach allows for compensating relative errors up to 40%, but presents an overall uncertainty estimated of the order of 5%. That value shall be added to the other causes of uncertainty resulting in the overall accuracy. The correction is required only for off axis sources and has been applied to both luminance and spectral measurements.

In the following, a brief description of the components used in the optical units is presented, showing the final achieved response in the visible-NIR range. The calibration procedures are then presented and the results of tests under different light sources are shown, reporting the overall errors on the final measured values.

### 3.1. Multi-Luminance Meter Calibration

**Figure 7:**
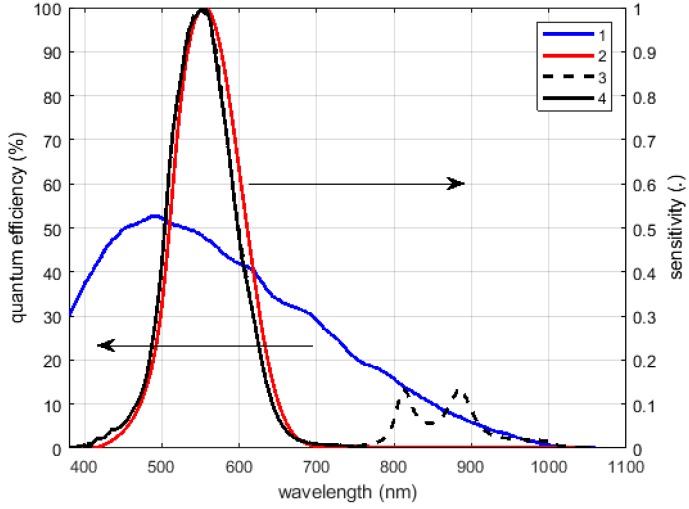
Camera efficiency (**1**), the normalized spectral sensitivities of the human eye (**2**), of the lens and the photopic filter (**3**), of the luminance meter with the near infrared (NIR) low-pass filter (**4**).

The image luminance meter is composed by a monochromatic camera, its lens, and a photopic filter; in order to calibrate the instrument according to the International System of Units, the overall system shall have a spectral sensitivity as close as possible to the standardized spectral sensitivity of the human eye V(λ)The required normalized spectral response versus the wavelengths (the visible range to near infrared) is presented using a red line in Figure 7 and, in the same figure, the response of the used camera sensor is plotted using a blue line.

The calibration of the multi-luminance meter was obtained under a luminance reference source: an integrating sphere with diameter of 38 mm available in the University Laboratory with a declared uncertainty equal to 2% of the set value. The luminance on the aperture presents a uniformity of 0.5% and its short-term stability is 0.5%. The current in the halogen lamp can be varied and was chosen to approximate the spectral emission of a blackbody at 2856 K, i.e., the Illuminant A. To have a more accurate control of the reference luminance, a spot luminance meter Konica Minolta LS-100 was used as reference in the calibration of the multi-luminance meter.

The measurements were collected using an exposure time multiple of 20 ms in order to correctly average the light emission over the repetition period of the power supply. The calibration coefficient was calculated by comparing the response of the multi-luminance meter and the reference luminance meter under the approximated Illuminant A with a luminance of 40 cd m^−2^.

The response of the camera plus the filter (Omega Optical Photopic Filter), neglecting the attenuation introduced by the lens, is presented in Figure 7 using a black dashed curve. The response of the obtained system is a good approximation of the photopic spectral sensitivity of the human eye in the visible range, where the maximum error is equal to 6% of the peak value. Above 750 nm, the response deviates significantly from the desired behavior so an additional NIR low-pass filter with cut off wavelength of 750 nm was added to correct the response at longer wavelengths. That correction avoids different responses of the system to the light coming from sources based on different technology, in particular for sources emitting also in the NIR region, like incandescent lamps. The final normalized spectral sensitivity of the realized multi luminance meter is represented in Figure 7 by the thicker back curve.

The multi luminance meter performance was investigated under different types of light sources: three fluorescent lamps, a High Pressure Mercury lamp, a high-pressure sodium lamp, and an incandescent lamp. The maximum calculated error is close to 6% of the reading, which is considered small enough for the application of light pollution monitoring. Table 2 presents the obtained luminance measures and the difference vs. the reference meter.

To quantifying the repeatability of the measured values, the standard deviation on samples of 10 camera measures were evaluated: the maximum calculated value is about 1% of the measured value.

The effect of the discrepancy between the spectral responsivity of the multi-luminance meter (*s_rel_*) and the spectral sensitivity of the human eye (*V*), when observing a light with a spectral distribution S_t_, can be quantified by using the color correction factor (*ccf*) [39]:

(1) $$ccf=\frac{\int_{\lambda_{min}}^{\lambda_{max}}S_{s}\left(\lambda \right)V\left(\lambda \right)d\lambda \cdot \int_{\lambda_{min}}^{\lambda_{max}}S_{t}\left(\lambda \right)s_{rel}\left(\lambda \right)d\lambda }{\int_{\lambda_{min}}^{\lambda_{max}}S_{s}\left(\lambda \right)s_{rel}\left(\lambda \right)d\lambda \cdot \int_{\lambda_{min}}^{\lambda_{max}}S_{t}\left(\lambda \right)V\left(\lambda \right)d\lambda },$$

where *S_S_* is the spectral distribution of the light used during calibration. From the definition of the color correction factor, it is apparent the correction can be applied only if the spectral measurement of the examined source is known. That required information can be provided by the multi-spectrometer.

The color correction factor was investigated to quantify the effect of the spectral error on the measurement of the luminance under the light of sources based on different technology. Table 3 shows the factors for the sources already presented in Table 2 and three colored LEDs. (The value 1 corresponds to no color error.)

As can be seen in Table 3, the color correction factor of the multi-luminance meter shows increased values for measurements under the colored LED sources, which have a narrower spectral power distribution than the other analyzed sources. It was considered of interest to analyze those colored light sources as they are often used in signs of commercial buildings and advertising billboards [40]; they also generate light pollution. For broad band sources like the ones used for outdoor lighting, the maximum error in ccf is around 8% for the HP sodium lamp.

### 3.2. Multi-Spectrometer Calibration

**Figure 8:**
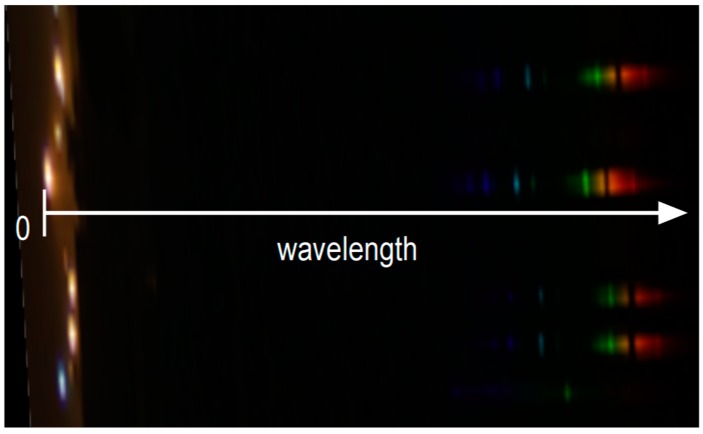
Light sources (on the left) and their spectra (on the right). Image obtained on a camera sensor by placing the diffracting grid in front of the lens. The picture is stretched along the vertical direction to allow a more apparent view of the spectra.

The multi-spectrometer uses the same monochromatic camera and the same lens as the multi luminance meter but is completed by a visible transmission diffracting grid with 300 groves/mm placed in front of the lens. When the camera frames a group of street light sources, its sensor records aside the image of each framed light source its spectral power density. An example is shown in Figure 8, where the image was recorded by an RGB camera for a better visual identification of the different emitting wavelengths, which are visible on the right. Furthermore, the picture is stretched along the vertical direction to allow a more apparent view of the spectra. The choice of a low value of the groves/mm of the diffracting grid reduces the side shift of the first order spectrum from zero order allowing the spectra of most of the framed sources to appear within the sensor.

In the image, the distance between the position of the source and a point in the image of the spectrum represents the wavelength, but is not proportional to it. As calculated for the multi-luminance meter, the sensitivity of the camera sensor depends on the wavelength and the same happens for the response of the diffracting grid. The values recorded by each pixel covered by the spectrum images are proportional to the power density at a specific wavelength, but the proportionality factor depends on the wavelength and a calibration of the amplitudes is therefore required.

To calibrate the wavelength scale, a High Pressure Mercury lamp was used, since its known emission lines are easily detectable. The expected Full Width at Half Maximum (FWHM) of the equivalent filter of the multi-spectrometer was 10 nm; therefore, each mercury line was spread in a finite wavelength interval, causing an uncertainty in the calibration process. To overcome this problem, the calibration of the wavelength axis was obtained comparing the multi-spectrometer data to the measurement from a calibrated spot spectrometer available in the laboratory (Konica Minolta CS-1000 with a spectral bandwidth of 5 nm and an uncertainty on the wavelength values equal to 0.3 nm). Ahead of the calibration procedure, the spectrum measured by the calibrated spot spectrometer was processed to obtain the same FWHM of the multi-spectrometer. The data from the multi-spectrometer were scaled considering the typical attenuation caused by the camera sensor and by the diffracting grid and finally compared with the measurement of the calibrated spot spectrometer. Consequently, the wavelength scale of the multi-spectrometer was adjusted minimizing the mean square difference between the measures of the power density provided by the two instruments. The wavelength scale of the multi-spectrometer was linearly interpolated between two peaks of the mercury lamp, and the power associated with each wavelength bin was evaluated considering power conservation.

**Figure 9:**
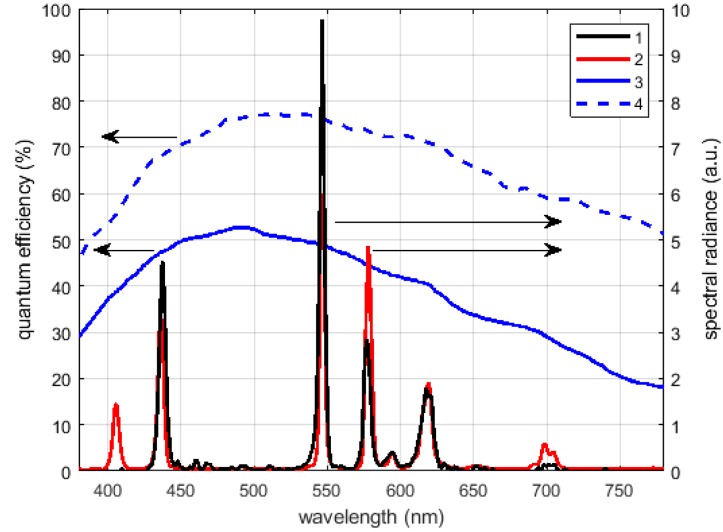
Distribution used for the wavelength calibration measured by the multi-spectrometer (1 black) and the Konica Minolta CS-1000 (2 red), and the amplitude values are normalized to the total received power. The camera sensitivity (3 continuous) and the diffracting grid attenuation (4 dashed) are in blue.

Figure 9 presents the results of the calibration process: the spectral radiance distribution provided by the two instruments, the camera sensitivity, and the attenuation of diffracting grid normalized to their maximum. It must be noted that the peak at about 406 nm shown by the CS-1000 is not detected by the multi-spectrometer; this is caused by a high attenuation introduced by the lens at the shortest wavelengths. In any case, this attenuation does not affect the luminance measurements significantly, since the sensitivity of the human eye and consequently of the photopic filter present very low values at those short wavelengths, as was shown in Figure 7.

The wavelength calibration can be considered to be effective, since a very good alignment of the peaks corresponding to the mercury lines is evident.

Figure 9 also shows that amplitude attenuations due to the camera and the diffracting grid are not well compensated at some wavelengths; this is mainly related to a very approximate knowledge of the diffracting grid behavior as declared in datasheets. 

Furthermore, both the equivalent bandwidth of the multi-spectrometer and the measured amplitudes of the spectrum are significantly influenced by the apparent size of the light source. In fact, the image of the spectrum in the acquired picture is the superposition of the contribution of each portion of the framed source and the effect may be considered negligible only for a source with an apparent size equal to one pixel (which can happen when flying at the altitude of few hundred meters but is extremely difficult to reproduce in the laboratory).

The correction factor required to adjust the spectrum amplitude was obtained directly comparing the measure of the multi-spectrometer and our reference. White Spectralon Diffuse Reflectance Standard was observed by the two instruments under a known light mix from an incandescent lamp and a white LED lamp, which provides a continuous spectrum providing enough power at every wavelength. The calibration of the spectral components at each wavelength was not performed as absolute measurement but normalized, since the measurements by the multi spectrometer will be used only to identify the source technology and to be able to correct the luminance measures through the color correction factor, if desired. Otherwise, the luminance multimeter will provide an estimation the absolute value of the luminance.

**Figure 10:**
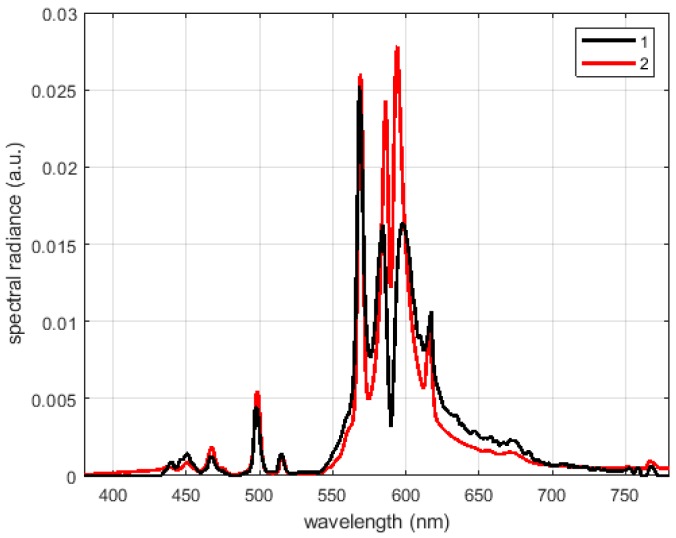
Distribution of a high pressure (HP) sodium lamp light measured by the multi-spectrometer (1 black) and the Konica Minolta CS-1000 (2 red).

As an example, the measured power density of a high-pressure sodium lamp is presented in Figure 10. The amplitude values are normalized to the total received power, i.e., the area under the measured spectrum. The two lines represent the measures from the multi-spectrometer (black line) and from the reference spectrometer (red line). The peaks of the line emission are well aligned even if the ones detected by the multi-spectrometer present lower amplitudes; higher values aside the peaks are direct consequences of the wider bandwidth of its equivalent filter.

### 3.3. Assessment of the Multi-Spectrometer

The final step of the calibration investigated the capability of the multi spectrometer in recognizing the emitted spectrum of an unknown source. The measured profile was compared with the one provided by the reference spectrometer Konica Minolta CS-1000, and the output was correlated using state-of-the-art techniques [41]. Among them, there is the correlation coefficient between two signals *x* and *y*, which is defined as

(2) $$\rho =\frac{Cov\left(x,y\right)}{\sigma_{x}\sigma_{y}}.$$

It is most frequently used for time signals, and the frequency response assurance criterion (FRAC), which is applied in the frequency domain [42] according to the following equation:

(3) $$FRAC=\frac{{\left|\sum_{f=f1}^{f2}X\left(f\right)Y^{*}\left(f\right)\right|}^{2}}{\sum_{f=f1}^{f2}X\left(f\right)X^{*}\left(f\right)\sum_{f=f1}^{f2}Y\left(f\right)Y^{*}\left(f\right)},$$

where *f*_1_ and *f*_2_ are the lower and upper frequency limits, respectively, and *X*(*f*) and *Y*(*f*) are the Fourier transform of the signals *x* and *y*; the symbol ‘∗’ denotes the complex conjugate.

Other correlation procedures were also considered [43,44] implying the separation of magnitude similarity (M_index_).

(4) $$M_{index}=\frac{1}{f_{2}-f_{1}}\int_{f_{1}}^{f_{2}}M_{\alpha }\left(f\right)df,$$

where

(5) $$M_{\alpha }=1-tanh\left(\frac{\ln3}{2}\frac{20\;log_{10}\left|\frac{Y\left(f\right)}{X\left(f\right)}\right|}{\alpha }\right),$$

where is a reference value in our case = 20, and the shape similarity (S_index_)

(6) $$S_{index}=\frac{1}{f_{2}-f_{1}}\int_{f_{1}}^{f_{2}}S_{\beta }\left(f\right)df,$$

(7) $$S_{\beta }=1-tanh\left(\frac{\ln3}{2}\frac{\left|\frac{1}{2\pi }\;\frac{d}{df}\left[arg\left(\frac{Y\left(f\right)}{X\left(f\right)}\right)\right]\right|}{\beta }\right),$$

where is a reference value in our case = 1, and the expression arg(A(*f*)) means the phase of the function A(*f*).

Some sources commonly used in street lighting were analyzed and the results obtained by comparing the data by multi-spectrometer and the reference spectrometer are presented in Table 4.

The high values of the correlation coefficient *ρ* show that the results of the multi-spectrometer are in good agreement with the ones of the reference spectrometer, apart from the case of the mercury lamp. The FRAC values describe the situation analogously, highlighting the lack of similarity for such kind of light. Lower values are apparent for FRAC, in particular with respect to the correlation coefficient; it is caused by the fact that FCRAC deals with squared quantities, as it appears in Equation (3); therefore, departures from the unitary value are emphasized. For a direct comparison, a column presents the square root of FRAC values; they are very close to ρ values. For all considered lamps, M_index_ values have a similar meaning that the amplitudes of the spectral density are recognized as being similar for all cases. The differences in the measures of the spectral density are so mainly related to the reconstruction of the shape of the emission spectrum causing the lower values of the S_index_. The mercury-based lamp presents many spikes in the spectrum, and the correction on the wavelength axis becomes more important: the wavelength residual misalignment corresponds to a shape variation, which is highlighted by the lower values of S_index_.

## 4. Discussion

Overall, the similarity indices confirm that the MINLU multi-spectrometer provides measures of the spectral density of the analyzed light with a quality close enough to the reference spectrometer available in the laboratory and thus can be considered effective in identifying the technology of the framed light sources. The cut off of the optics at the shortest wavelength is a small lack, luckily it does not affect the ability in identifying neither white LEDs nor blue colored LEDs. In both cases, the peak at short wavelengths is very far from 406 nm, being between 440 nm and 450 nm for the white LEDs and at about 470 nm for a blue LED. Future investigation could be devoted to analyzing what is the real lower limit of the multi-spectrometer.

The data collected by the multi spectrometer can be used to calculate directly G-index [45]. The value of this parameter is directly related to blue light content, light power at the shortest wavelengths; therefore, it provides a quantification of the impact of the considered light to light pollution and of how much it can affect wildlife during night and star visibility. Besides the standards devoted to outdoor and road safety [46,47,48,49,50], recommendations were prepared and studies were performed dealing with energy consumption, environmental impact of outdoor lighting, light pollution, and impact of lighting on wildlife and road management systems [51,52,53]. Significant aspects of the impact of artificial lighting at night depend on the spectral power distribution of light; G-index was included in various documents aiming at reducing things that are not worth using and dangerous blue content of outdoor lighting.

In the MINLU system, the luminance meter and the spectrometer are also able to deal with colored light; in particular, the performance of the multi luminance meter was highlighted considering colored LEDs. That ability can be useful during flying inspections; signs of commercial buildings, advertising billboards, and wide self-luminous building façades are more and more often made by using colored RGB LEDs; all those light sources generate light pollution and shall be dealt with; it is possible by the MINLU instrument.

MINLU can include a Stellarnet Black Comet; the performance of that spectrometer far exceeds the ones of the multi-spectrometers. It allows for measuring the total upward light, but it is not able to discriminate the contribution of the different light sources. Furthermore, it can not distinguish the light emitted from primary sources from the light reflected from lit surfaces. On the contrary, the multi-spectrometer allows for selecting each primary source, but it can not deal with light reflected by lit surfaces. Their presence is complementary.

An absolute quantification of the spectrum of the light is not obtained by the presented calibration; therefore, it is not easy to compare the performance of the multi-spectrometer with commercial instruments. The performance of these last ones is commonly quantified under Illuminant A and poor information describes their spectral behavior. The performance of the multi-luminance meter can be compared with standards [54,55]. Usually, the luminance meters are calibrated under Illuminant A, approximated by a source based on a halogen lamp emitting at 2856 K. The calibration uncertainty is defined under that source. For the multi-luminance meter, this uncertainty is about 2%. The estimated spectral mismatch index *f*1′ [56] is equal to 6%, which is confirmed by the ccf values of Table 3. Considering those parameters and an overall uncertainty less than 10% of the measured value, see Table 2; according to UNI [54] and to DIN [55] standards, the multi-luminance meter belongs to the class B and can be considered a good photometer [3].

Digital single-lens reflex (DSLR) cameras can be used to collect images from drones or tethered balloons. Often, the luminance values and the identification of the kind of light sources are obtained by a combination of the three images corresponding to the R, G, and B filters. In the MINLU setup, the construction of a luminance meter using a monochromatic camera and a photopic filter allows a better spectral response. The introduction in MINLU of a multi-spectrometer allows the identification of spot light sources and their spectrum and technology. RGB DSLR camera can have a better spatial resolution, but the kind of the analyzed lamps has to be deduced from the values of the power in the three R, G, and B bands, it reduces their discriminating ability. The commercial low-cost monochromatic cameras used in MINLU allow an easy acquisition through an analog to digital converter with only 12 bits; DSRL cameras usually have a wider dynamics resolution of 14 bit allowing better details recognition.

Table 5 resumes the main positives and threats of the MINLU system.

## 5. Conclusions

The calibration of the proposed image suite allows the measurements of the luminance and the power spectral density of the framed emitting sources with an uncertainty comparable with that of industrial instruments. According to the evaluated performance, the multi-luminance meter belongs to class B of the UNI and DIN standards. In particular, in the worst case, the error on the luminance measurement of the analyzed white light sources is less than 7% of the measured value (Table 2) and the spectral error of the multi-luminance meter is no more than 8% for “white” light (Table 3). Other parameters can be analyzed and will be considered in the future; some of them are presented in Annex F of [53]. Among them, a part the parameters cited above, the spectral mismatch of the multi-luminance meter was also estimated; it is about 6%. This value is not as good as what is required to collect data of lamps and luminaires (*f*1′ = 2%), according to European standards [57]. There are requisites for instruments employed in laboratories, MINLU instruments will work in more rough conditions, and the authors consider that the obtained performance should be enough for their issues. In fact, the multi-luminance meter and the multi-spectrometer allow for identifying polluting sources, their type, and to quantify their emission.

In more detail, the multi-spectrometer included in the system has been tested to have a wavelength resolution of 2 nm and a discrimination capability of 10 nm significantly overcoming the performance achievable by measures obtained by satellites in discriminating among the different light sources used for street and outdoor lighting on the basis of the measurement of their power density.

The limited dimensions and weight of the unit permit the use on drones and tethered balloons to analyze upward emissions on a wide area and over several hours during the night, following the dimming of outdoor and street lights.

First outdoor measurements were performed by high buildings, the framed images allow for identifying outdoor light sources well and measuring their luminous intensity. Even if the pixel depth is not high, measurements of lit surfaces, e.g., road paving and building facades, were done. The acquired data will be compared with luminance measures obtained by other multi-luminance meters. A detailed analysis of those preliminary data will be performed in the future. The first test flight is under preparation. It will allow for understanding how the images of the framed light sources are influenced by the motion of the drone or balloon and how many ambient conditions affect the quality of the measures.

## Figures and Tables

**Table 1:** Main specifications of the monochromatic and color Basler cameras.

Camera	acA3088-16gm	acA3088-16gc
Sensor name	IMX178LLJ-C	IMX178LQJ-C
Sensor type	CMOS	
shutter	rolling	
mono/color	Mono	color
Resolution (H × V Pixels)	3088 × 2064	
Optical size	1/1.8”	
Effective Sensor Diagonal	8.92 mm	
Pixel Size (H × V)	2.4 µm × 2.4 µm	
Image Data Interface	Gigabit Ethernet (1000 Mbit/s)	
A/D converter	12 bits	

**Table 2:** Luminance of a white target lit by lamps based on different technology: the spot luminance meter Konica Minolta LS-100 is used as a reference.

Lamp	Ref. (cd m^−2^)	Multi-Lum. (cd m^−2^)	Diff. (%)
Fluorescent 2600 K	10.0	9.99	−0.1
Fluorescent 3750 K	11.4	11.2	2.1
Fluorescent 5800 K	10.7	11.1	−3.9
High Pressure Mercury	54.3	57.8	6.4
HP Sodium	55.0	52.3	−4.9
Incandescent	12.7	12.4	−2.3

**Table 3:** Color correction factor (ccf) of the multi-luminance meter and the spot luminance meter LS-100 used as reference.

Lamp	ccf	
	Multi_lum.	LS-100
Fluorescent 2600 K	1.030	1.014
Fluorescent 3750 K	0.995	1.021
Fluorescent 5800 K	0.961	1.030
High Pressure Mercury	0.996	1.005
HP Sodium	1.082	0.960
Incandescent	1.007	0.997
Red LED	1.317	0.970
Green LED	0.848	1.027
Blue LED	0.755	1.097

**Table 4:** Similarity indices comparing the performances of the multi-spectrometer and the reference spectrometer Konica Minolta CS-1000, for common street lamps based on different technology.

Lamp	Corr. Coeff.	*p*	FRAC	$\sqrt{\mathrm{FRAC}}$	M_index_	S_index_
Fluorescent 2600 K	0.943	<<0.05	0.907	0.952	0.870	0.636
Fluorescent 3750 K	0.828	<<0.05	0.744	0.863	0.845	0.460
Fluorescent 5800 K	0.832	<<0.05	0.761	0.872	0.873	0.472
High Pressure Mercury	0.605	<<0.05	0.416	0.645	0.872	0.547
HP Sodium	0.894	<<0.05	0.839	0.916	0.872	0.871
LED PC Amber	0.998	<<0.05	0.997	0.998	0.989	0.982

**Table 5:** Main positives, threats and limits of the proposed instrument.

Pros	Cons and Limits
Low cost of the materials	Medium pixel depth (12 bit)
Good spatial resolution	Mechanical delicacy of the optic components
Good spectral response for white light	Flying by night required special permissions
Direct measurement of the spectrum of polluting sources	Flying a drone by night required a special license
Good identification of the kind of polluting sources	MINLU weighs about 2.5 kg
Simple integration with the acquiring system	25 min of flight duration when held by a drone
MINLU allows the analysis of a significant part of the night when held by a tethered balloon	4 h maximum MINLU operating time
